# The Amino Acid Substitution Q65H in the 2C Protein of Swine Vesicular Disease Virus Confers Resistance to Golgi Disrupting Drugs

**DOI:** 10.3389/fmicb.2016.00612

**Published:** 2016-04-27

**Authors:** Ángela Vázquez-Calvo, Flavia Caridi, Mónica González-Magaldi, Juan-Carlos Saiz, Francisco Sobrino, Miguel A. Martín-Acebes

**Affiliations:** ^1^Centro de Biología Molecular Severo Ochoa (CSIC-UAM)Madrid, Spain; ^2^Departamento de Biotecnología, Instituto Nacional de Investigación y Tecnología Agraria y Alimentaria (INIA)Madrid, Spain

**Keywords:** *enterovirus*, Golgi, brefeldin A, replication complex, resistance, mutant

## Abstract

Swine vesicular disease virus (SVDV) is a porcine pathogen and a member of the species *Enterovirus B* within the *Picornaviridae* family. Brefeldin A (BFA) is an inhibitor of guanine nucleotide exchange factors of Arf proteins that induces Golgi complex disassembly and alters the cellular secretory pathway. Since BFA has been shown to inhibit the RNA replication of different enteroviruses, including SVDV, we have analyzed the effect of BFA and of golgicide A (GCA), another Golgi disrupting drug, on SVDV multiplication. BFA and GCA similarly inhibited SVDV production. To investigate the molecular basis of the antiviral effect of BFA, SVDV mutants with increased resistance to BFA were isolated. A single amino acid substitution, Q65H, in the non-structural protein 2C was found to be responsible for increased resistance to BFA. These results provide new insight into the relationship of enteroviruses with the components of the secretory pathway and on the role of SVDV 2C protein in this process.

## Introduction

Swine vesicular disease virus (SVDV) is a member of the species *Enterovirus B* within the *Picornaviridae* family. SVDV is the etiological agent of a highly contagious disease of pigs (SVD) and is closely related to the human pathogen coxsackievirus B5, CVB5 (Zhang et al., 1999; Bruhn et al., 2015). As other RNA viruses, enterovirus populations are quasispecies and exhibit a high potential for variation and adaptation, including the rapid selection of drug resistant variants (Andino and Domingo, 2015).

SVDV genome is composed of a single-stranded RNA molecule of positive polarity that is translated using an internal ribosome entry site (IRES) to produce a single polyprotein that is processed into the mature viral proteins (Zhang et al., 1993; Escribano-Romero et al., 2000). The four structural proteins (VP1-VP4) form an icosahedral capsid of approximately 25–30 nm in diameter and the seven non-structural proteins (2A, 2B, 2C, 3A, 3B, 3C, and 3D) are involved in the virus life cycle (Whitton et al., 2005; Buenz and Howe, 2006). Positive-strand RNA viruses induce a dramatic remodeling of the intracellular membranes of infected cells aimed to develop an adequate environment for RNA replication (Hsu et al., 2010; Harak and Lohmann, 2015). In fact, these structures are considered specialized organelles for viral replication (Hsu et al., 2010; Richards et al., 2014). In enteroviruses, such as SVDV, RNA replication takes place at the surface of cytoplasmic vesicular structures derived from cellular membranes of the endoplasmic reticulum (ER) and the Golgi complex (Martín-Acebes et al., 2008; Belov and Sztul, 2014; Linden et al., 2015). Studies mostly performed with poliovirus (PV) have shown that the enteroviral proteins 2B, 2C, and 3A are involved on membrane rearrangements (Bienz et al., 1994; Suhy et al., 2000; Belov et al., 2008; Hsu et al., 2010), albeit the mechanisms of membrane reorganization are not well understood yet. In some cases, the proposed mechanism of action of these non-structural proteins involves their interaction with host cell proteins from the secretory pathway, which can vary among picornaviruses (Gazina et al., 2002; Martín-Acebes et al., 2008; van der Linden et al., 2010; Sasaki et al., 2012).

Brefeldin A (BFA) is a fungal metabolite that inhibits the replication of several enteroviruses, including PV and SVDV (Maynell et al., 1992; Crotty et al., 2004; Martín-Acebes et al., 2008; van der Linden et al., 2010; Viktorova et al., 2015). BFA blocks the function of the cellular protein ADP ribosylation factor 1 (Arf1) through the inhibition of several guanine nucleotide exchange factors (GEFs) that regulate its activity, such as GBF1 (Golgi-specific BFA resistance factor 1), BIG1 (BFA-inhibited GEF 1) and BIG2 (Klausner et al., 1992; Chardin and McCormick, 1999). Arf1 is localized on the Golgi complex and the ER-Golgi intermediate compartment. Its activated form induces formation of secretory vesicles via recruitment of effector proteins such as the coatomer protein complex I (COP-I) during vesicle budding (D'souza-Schorey and Chavrier, 2006; Donaldson and Jackson, 2011). Golgicide A (GCA) is also an inhibitor of GBF1 (Pan et al., 2008; Sáenz et al., 2009). Despite BFA and GCA induce fragmentation of the Golgi complex by inhibition of Arf1 activation, they can differentially affect picornavirus replication (Martín-Acebes et al., 2008; van der Linden et al., 2010). The differences in the sensitivity to Golgi disrupting drugs support that diverse cellular factors are required among picornaviruses to form the replication complex. Thus, BFA and GCA have become useful tools to analyze the requirements for picornavirus replication (Gazina et al., 2002; Martín-Acebes et al., 2008; van der Linden et al., 2010; Mousnier et al., 2014). In this context, PV variants resistant to BFA show point mutations associated to resistance that are located in the 2C and 3A proteins (Crotty et al., 2004; Viktorova et al., 2015).

In this study, we show that BFA and GCA inhibit SVDV replication. Furthermore, the analysis of BFA-resistant SVDV mutants revealed that a single amino acid substitution in 2C protein was responsible for the increased resistance to BFA. These results provide new insight into the relationship of enteroviruses with the components of the secretory pathway and on the role of SVDV 2C protein in these processes.

## Materials and methods

### Cells, viruses, reagents, and antibodies

IBRS-2 (De Castro, 1964) and BHK-21 cells (ATCC) were maintained in Dulbecco's modified Eagle's medium (DMEM) (Gibco) supplemented with 5% fetal calf serum (FCS) (Gibco), l-glutamine (2 mM), penicillin (100 U/ml) and streptomycin (100 μg/ml). The SVDV isolate used in this study, SPA/1/′93 (Jiménez-Clavero et al., 1998), was propagated on IBRS-2 cells. Viral stocks of type C foot-and-mouth disease virus (FMDV) C-S8c1 (Sobrino et al., 1983) and vesicular stomatitis virus (VSV) Indiana (Vázquez-Calvo et al., 2011) were prepared by amplification in BHK-21 cells. BFA and GCA (Sigma) were dissolved in dimethyl sulfoxide (DMSO). Control cells were treated in parallel with the same amount of drug vehicle. A rabbit polyclonal antiserum against CVB3 2C protein was utilized for SVDV protein detection (Martín-Acebes et al., 2008). *Cis*-Golgi networks was stained using MAb 25H8 to gp74 (Alcalde et al., 1994) or a MAb to the C-terminal region of GM130 (ECM Biosciences). Doubled-stranded RNA (dsRNA) was detected using a MAb J2 (English & Scientific Consulting). Alexa Fluor 488 (green) and 594 (red) conjugated anti-mouse or anti-rabbit secondary antibodies (Invitrogen) were used to recognize primary antibodies in immunofluorescence assays.

### Cell viability assay

The cytotoxicity of the drugs was determined by measuring the cellular ATP using the CellTiter-Glo Luminiscent Cell Viability assay (Promega). IBRS-2 cells were seeded in 96-well plates, incubated with increasing concentrations of the drugs for 24 h, and assayed as recommended by the manufacturer.

### Immunofluorescence

Cells grown on glass coverslips were washed with PBS, fixed with 4% paraformaldehyde or with methanol for gp74 staining, blocked, and permeabilized with PBTG buffer (0.1% Triton X-100, 1% bovine serum albumin [BSA], and 1M glycine in PBS) for 15 min at room temperature. Samples were incubated with primary antibodies diluted in 1% BSA in PBS for 1 h at room temperature, washed with PBS, and incubated with fluorescently labeled secondary antibodies for 30 min at room temperature, as described (García-Briones et al., 2006). After washing with PBS, nuclei were stained with 1 μg of DAPI (4′,6′-diamidino-2-phenylindole; Invitrogen)/ml or with To-Pro-3 (Molecular Probes). Samples were mounted in Fluoromount G (Southern Biotech) and observed with an Axioskop microscope (Zeiss) coupled to a digital monochrome camera coolsnap FX (Roper Scientific). Images were acquired by using RS Image 1.9.2 software (Roper Scientific) and processed using Adobe Photoshop CS2 (Adobe Systems, Inc.). For confocal laser scanning microscopy, samples were observed using a Leica TCS SPE confocal lases scanning microscope. Images were acquired using Leica Advanced Fluorescence Software and processed with Adobe Photoshop CS2 (Adobe Systems, Inc.). Optical slice thickness for all confocal images displayed was of 1 airy unit.

### Infections and virus titrations

Triplicate wells of IBRS-2 cells pretreated or not with the drugs for 30 min were infected with VSV, SVDV, or FMDV using a multiplicity of infection (moi) of 0.5 plaque forming units (pfu)/cell. After the first infection hour, viral inoculum was removed and fresh medium containing or not the drug and 5% FCS was added (this time point was considered 1 h postinfection [pi]). Eight hours later, cells were subjected to three freeze-thaw cycles, and the total (intracellular and medium-released) virus yield was determined by plaque assay (in BHK-21 cells for VSV and FMDV or in IBRS-2 cells for SVDV), as described (Martín-Acebes et al., 2009).

### Isolation of BFA-resistant SVDV mutants

IBRS-2 cells were infected with SVDV in the presence of 0.5 μg/ml BFA and when total cytopathic effect was observed, cells were subjected to three freeze-thaw cycles to harvest the progeny virus (termed B_1_ population) and virus yield was determined by plaque assay. This procedure of infection was repeated five additional times in the presence of increased concentrations of BFA (1, 1.5, 2, 2.5, and 3 μg/ml) to produce viral populations B_2_-B_6_. All serial passages were performed with a moi of 0.01 pfu/cell. The isolation of the nine BFA-resistant clones was performed by biological cloning in IBRS-2 cells infected with SVDV B_6_ population in semisolid agar medium containing 3 μg/ml of BFA. For this purpose, viruses from well isolated lysis plaques (24 h pi) were picked and amplified by infection in liquid medium containing 3 μg/ml of BFA.

### Viral RNA extraction, CDNA synthesis, and DNA sequencing

Viral RNA was extracted from supernatants of infected cell cultures by using TRI Reagent (Sigma). cDNA synthesis and DNA sequencing were performed as described (Vázquez-Calvo et al., 2016). Nucleotide positions correspond to those reported for the SVDV SPA/1/′93 isolate (GenBank KU291213).

### Data analysis

Analysis of the variance (ANOVA) using *F* Fischer–Snedecor distribution was performed with statistical package SPSS 19.0 (SPSS Inc.). One asterisk (^*^) or two asterisk (^**^) in the figures denote statistically significant differences with *P* < 0.05 or *P* < 0.005, respectively.

## Results

### Effect of golgi complex disrupting agents on SVDV infection

**Figure 1:**
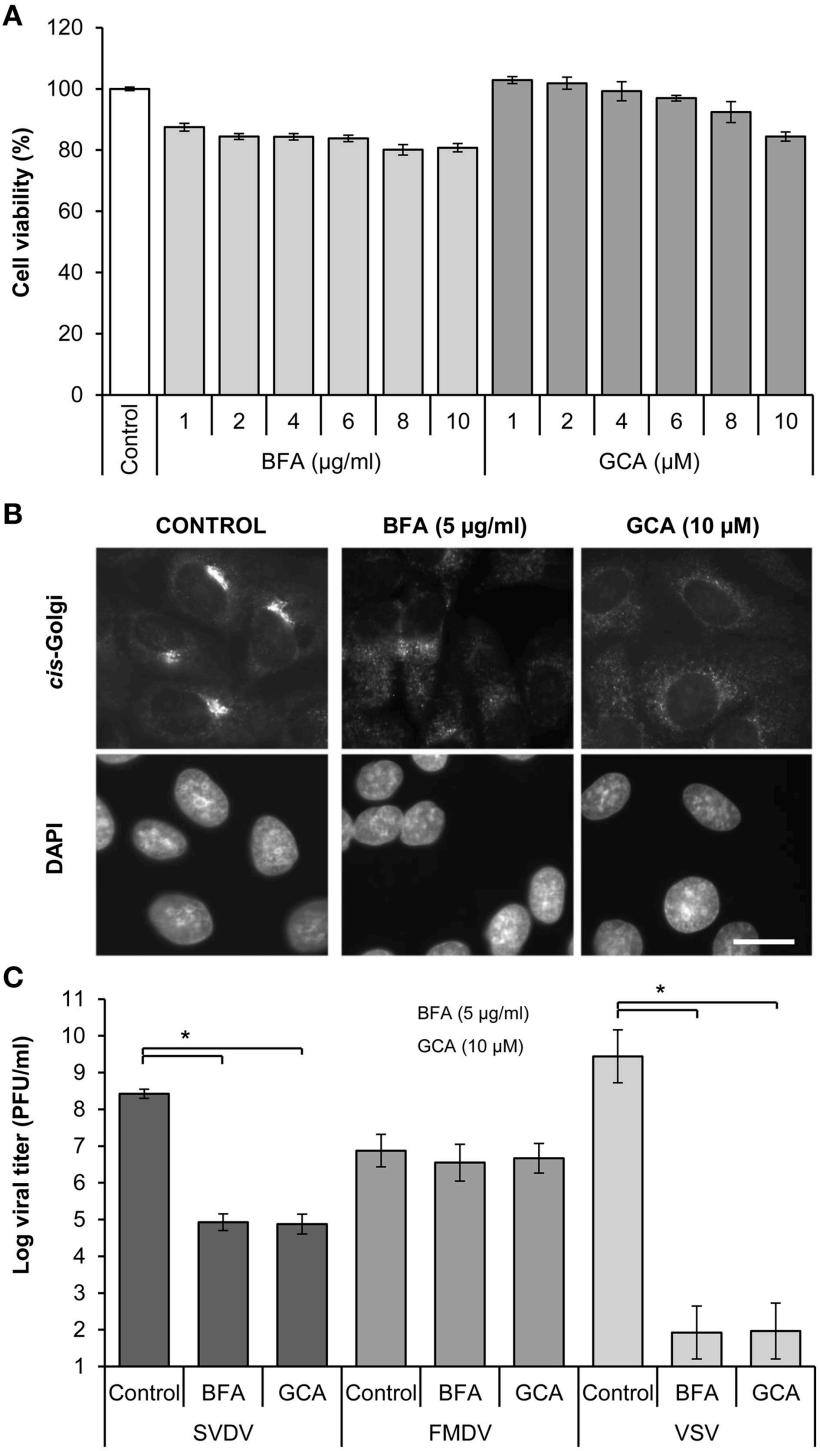
**Effect of Golgi disrupting drugs on SVDV, FMDV, and VSV multiplication. (A)** Effect of BFA (1, 2, 4, 6, 8, or 10 μg/ml) and GCA (1, 2, 4, 6, 8, or 10 μM) on IBRS-2 cell viability determined by ATP measurement. **(B)** IBRS-2 cells were treated with DMSO (control), BFA (5 μg/ml) or GCA (10 μM) for 30 min. Cells were fixed and processed for immunofluorescence using a MAb against the *cis*-Golgi. Nuclei were stained using DAPI. Bar: 20 μm. **(C)** IBRS-2 cells treated as in **(B)** were infected with the corresponding virus (moi of 0.5 pfu/cell) in presence of the drug. At 8 h pi the viral titer was determined by plaque assay. Error bars represent SD. The data presented correspond to three independent experiments. Data were analyzed using ANOVA. Asterisk (^*^) denote statistically significant differences with *P* < 0.05.

We first assessed the cytotoxicity of two different Golgi disrupting agents, BFA and GCA, on IBRS-2 cells. None of the drug concentrations tested exerted major cytotoxic effect on IBRS-2 cells after 24 h of treatment, being the amount of cellular ATP higher than 80% of that of control cells in all cases (Figure 1A). However, immunofluorescence analyses revealed that treatment of the cells with these drugs caused the dispersion of a *cis*-Golgi complex marker through the cytoplasm of the cells (Figure 1B), which confirmed the expected effect of these Golgi disrupting agents. Next, the effect of these drugs on the infection of SVDV was evaluated. FMDV, a picornavirus that is resistant to BFA (Martín-Acebes et al., 2008), and the rhabdovirus VSV, which is highly sensitive to Golgi disrupting agents (Martín-Acebes et al., 2008), were included as controls (Figure 1C). As expected (O'Donnell et al., 2001; Martín-Acebes et al., 2008; Midgley et al., 2013), no statistically significant reductions in FMDV titers were found, whereas VSV production was inhibited by the two drugs tested. These results indicate that, as described for the enterovirus CVB3 (van der Linden et al., 2010), SVDV is inhibited by BFA and GCA.

### Isolation of BFA-resistant SVDV mutants

**Figure 2:**
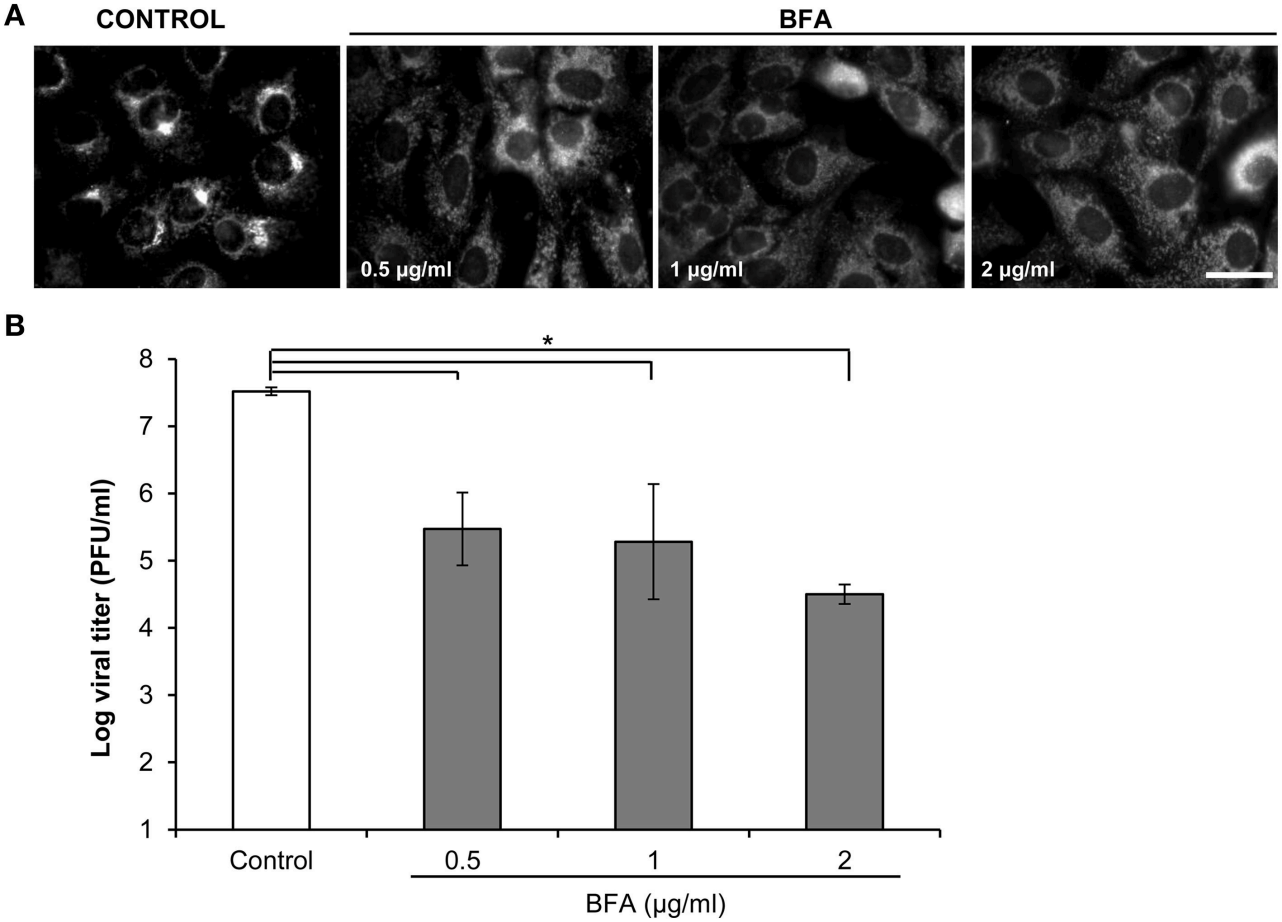
**BFA inhibits SVDV production in a dose-dependent manner. (A)** IBRS-2 cells treated with different concentrations of BFA for 30 min were fixed and processed for immunofluorescence using a MAb against the *cis*-Golgi. **(B)** IBRS-2 cells treated with BFA as in **(A)** were infected with SVDV (moi of 0.5 pfu/cell) in presence of the drug. At 8 h pi the viral titer was determined by plaque assay. Error bars represent SD. The data presented correspond to two independent experiments. Data were analyzed using ANOVA. Asterisk (^*^) denote statistically significant differences with *P* < 0.05.

**Figure 3:**
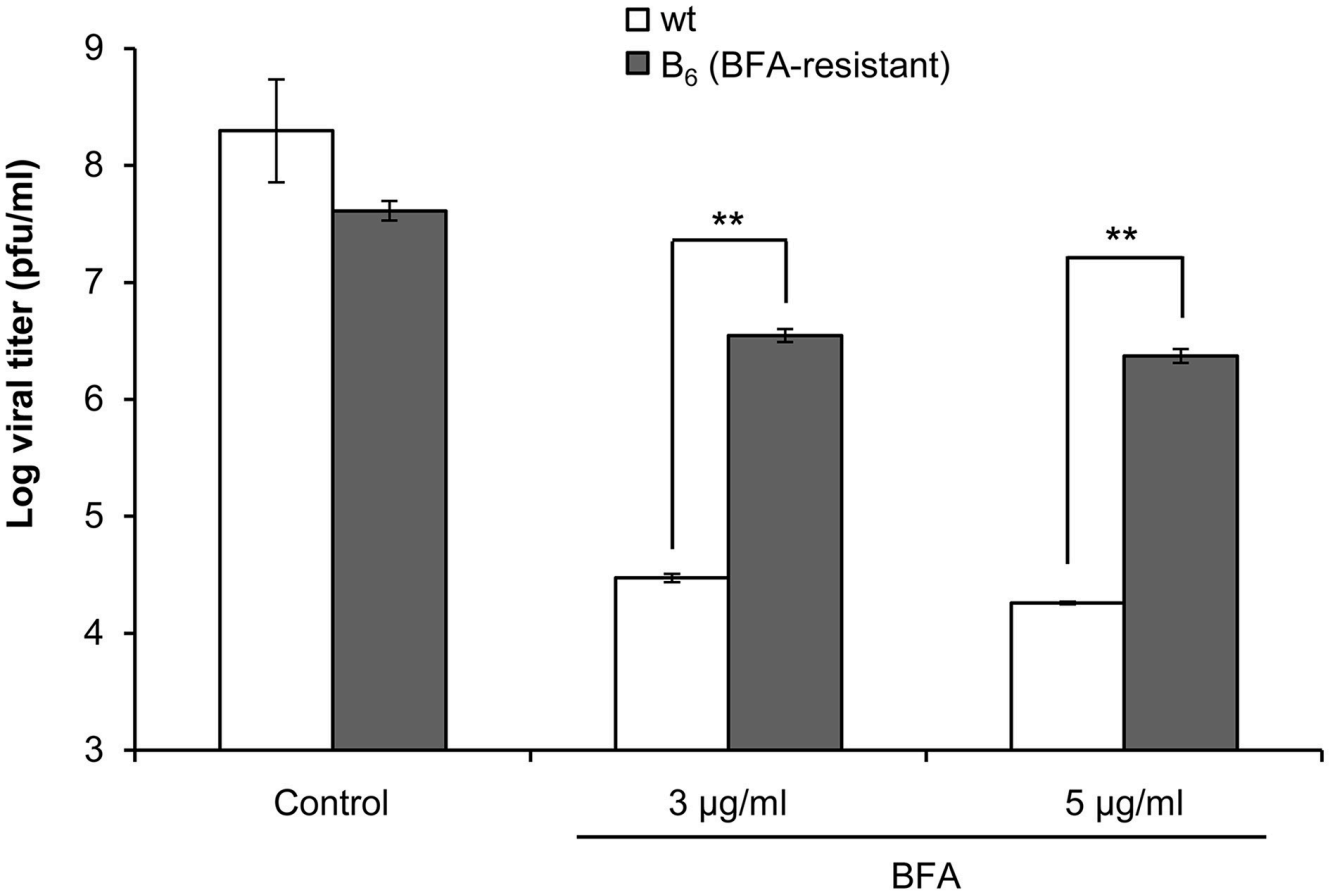
**SVDV B_6_ is more resistant to BFA than SVDV wt**. IBRS-2 cells treated with different concentrations of BFA for 30 min were infected with SVDV wt or SVDV B_6_ (moi of 0.5 pfu/cell) in presence of the drug. At 8 h pi the viral titer was determined by plaque assay. Error bars represent SD. Data were analyzed using ANOVA. Two asterisks (^**^) denote statistically significant differences with *P* < 0.005.

A valid strategy to gain information on the viral factors involved in the resistance to Golgi-disrupting agents such as BFA consists of the selection and characterization of mutants upon virus passages in the presence of increasing drug concentrations (Crotty et al., 2004; Viktorova et al., 2015). As shown in Figure 1, 5 μg/ml BFA caused disruption of the Golgi complex and inhibited SVDV yield by several orders of magnitude. To search for conditions for selection of BFA resistant mutants, the effect of lower drug concentrations was determined. Treatment with 0.5 μg/ml BFA was sufficient to disrupt the Golgi complex in IBRS-2 cells (Figure 2A) producing a statistically significant reduction (about two orders of magnitude) in the viral titer (Figure 2B). Then, SVDV was subjected to 6 serial passages in the presence of increasing concentrations of BFA, starting from 0.5 μg/ml up to 3 μg/ml in passage 6 (Table 1). During these passages viruses were harvested when total cytopathic effect was observed (usually about 24–40 h pi.). These viral populations were termed B_1_ to B_6_. In these experiments, we observed that although infections were performed using the same moi in each passage, the viral titer increased with the passage number, which was consistent with the selection of BFA-resistant viruses. To test whether the B_6_ population displayed increased resistance to BFA, IBRS-2 cells were infected in presence of different concentrations of the drug and the viral titers determined were compared with those from cells infected with the initial SVDV population, termed wt. The virus yield displayed by the wt virus was similar or even higher to that of B_6_ population in the absence of the drug (Figure 3). Conversely, the virus titers found in the viral population B_6_ grown in the presence of 3 or 5 μg/ml BFA were about two orders of magnitude higher than those of the wt. These results confirmed that SVDV population B_6_ showed increased resistance to BFA.

**Table 1:** **SVDV passages in presence of BFA**.

**Virus population^a^**	**BFA (μg/ml)**	**TCE^b^ (h pi)**	**Viral titer (pfu/ml)**
Wt	0.0	18	1.4 × 10^8^
B_1_	0.5	36	5.9 × 10^6^
B_2_	1.0	40	3.8 × 10^6^
B_3_	1.5	43	8.9 × 10^5^
B_4_	2.0	21	4.9 × 10^7^
B_5_	2.5	24	6.0 × 10^7^
B_6_	3.0	24	6.4 × 10^7^

a *Virus passaged in the presence of increasing concentrations of BFA. A moi of 0.01 was used for each passage*.

b *Time pi at which total cytopathic effect was observed (TCE)*.

### The amino acid substitution Q65H in the SVDV 2C protein confers resistance to BFA

**Figure 4:**
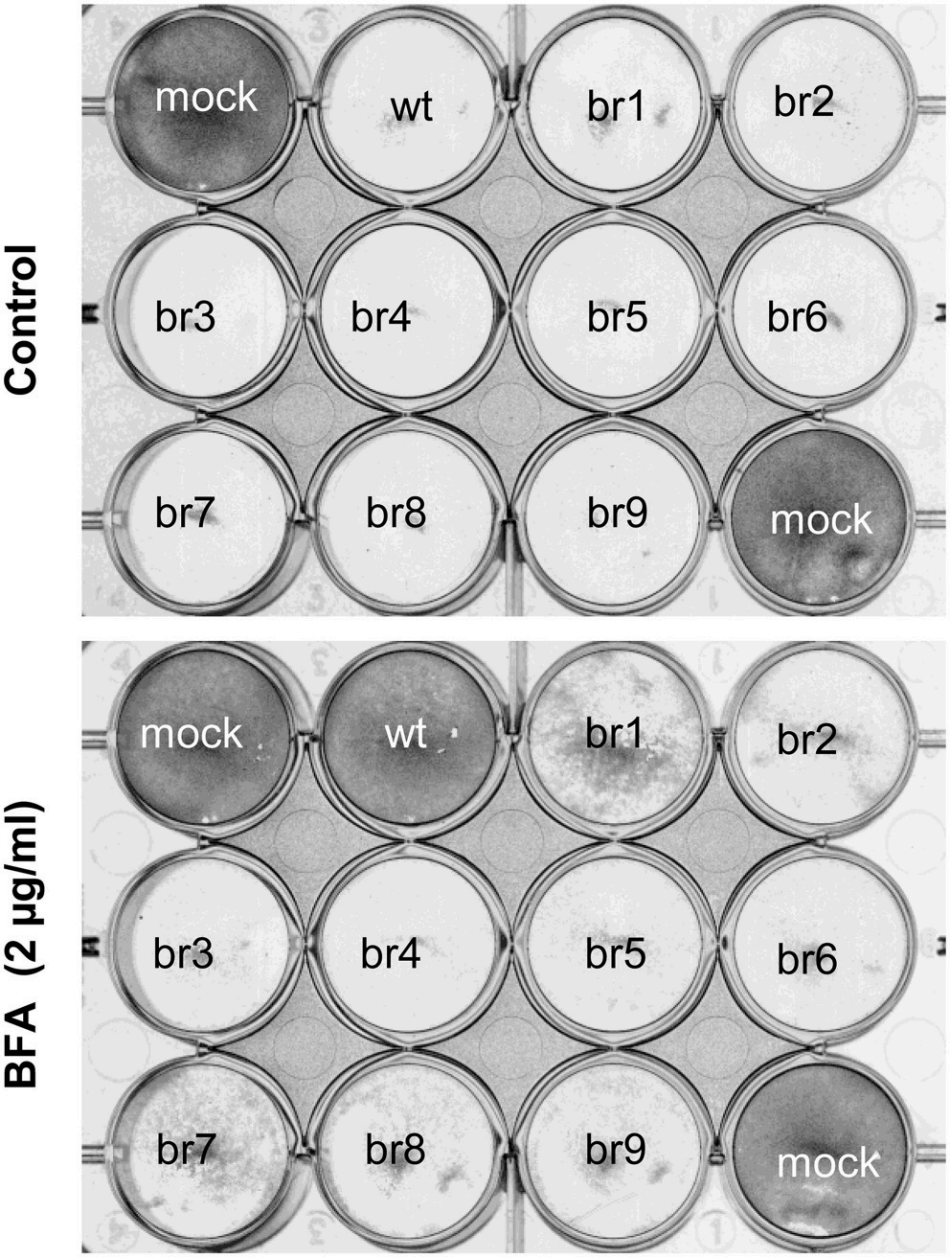
**Susceptibility of clones derived from SVDV B6 to BFA**. IBRS-2 cells were infected with SVDV wt or the different drug resistant br1-9 clones at a moi of 0.1 pfu/cell and then incubated for 40 h in the absence or presence of 2 μg/ml BFA.

To identify the genotypic changes responsible for the increased resistance to BFA in the SVDV population B_6_, the complete coding region sequences of the wt and B_6_ SVDV populations were determined and compared. The analysis of chromatograms of B_6_ population showed a single non-synonymous nucleotide replacement, G3659T, imposed, leading to amino acid replacement Q65H in the 2C protein (Table 2). Two additional positions with nucleotide mixture were also observed in B_6_ population: A2269A/G in a 42/58 ratio (G leading to substitution VP1 Q134R) and T4807T/C in a 53/47 ratio (C leading to replacement 3C V8A). To confirm the imposition of replacement 2C Q65H (G3659T) nine biological clones (termed br1-9) isolated from cells infected with B_6_ population and maintained in a semi-solid medium containing BFA, were amplified by a single infection in liquid medium in presence of BFA (3 μg/ml). Nucleotide sequencing of the 2C-3A coding regions (Table 2) confirmed that replacement 2C Q65H was present in all clones analyzed (br1-9). Virus br1 and br4 showed an additional synonymous change (A4575G), and virus br8 exhibited an additional non-synonymous nucleotide change G3610A, leading to replacement R49K in 2C protein. Changes A2269G (VP1 Q134R) and T4807C (3C V8A) partially imposed in the parental B_6_ population were found in the RNA of four (br2, br5, br7, and br9) and two (br3 and br6) of the clones analyzed, respectively. These results indicate that 2C Q65H, the only replacement found in the nine clones analyzed, is likely to be responsible of the increased resistance to BFA displayed by the B_6_ population. Next, the growth of br1-9 clones in the presence of BFA was analyzed (Figure 4). At low moi (0.01 pfu/cell), while the wt virus lysed the cells in the absence of drug, it did not produce detectable cytopathic effect in the presence of BFA. On the contrary, viruses br1-9 produced a similar cytopathic effect in both situations, which confirms that replacement 2C Q65H confers SVDV resistance to BFA.

**Table 2:** **Mutations found in the genome of SVDV mutants with increased resistance to BFA**.

**Genomic region**	**Mutations found in viral population^a^**									
	**B_6_**	**br1**	**br2**	**br3**	**br4**	**br5**	**br6**	**br7**	**br8**	**br9**
VP1	A2269A/G Q134Q/R^b^		A2269G **Q134R**			A2269G **Q134R**		A2269G **Q134R**		A2269G **Q134R**
2C									G3610A **R49K**	
	G3659T **Q65H**	G3659T **Q65H**	G3659T **Q65H**	G3659T **Q65H**	G3659T **Q65H**	G3659T **Q65H**	G3659T **Q65H**	G3659T **Q65H**	G3659T **Q65H**	G3659T **Q65H**
3A		A4575G			A4575G					
3C	T4807T/C V8V/A^b^		T4807C **V8A**	T4807C **V8A**		T4807C **V8A**	T4807C **V8A**	T4807C **V8A**		T4807C **V8A**

a *B_6_ refers to viral population recovered after 6 passages in the presence of increasing concentrations of BFA (see Material and Methods for details). br1 to br9 refer to independent biological clones isolated from B_6_. The complete coding region of B_6_ population was sequenced. Only genomic regions showing nucleotide replacements are included in the Table. For clones br1-br9, only the regions where B_6_ populations exhibited nucleotide substitutions were sequenced. The nucleotide position in the SVDV SPA/1/′93 genome (GenBank KU291213) and the substitution found are indicated. For non-synonymous substitutions, amino acid replacements are shown in bold*.

b *Nucleotide mixture at this position. See the text for details*.

**Figure 5:**
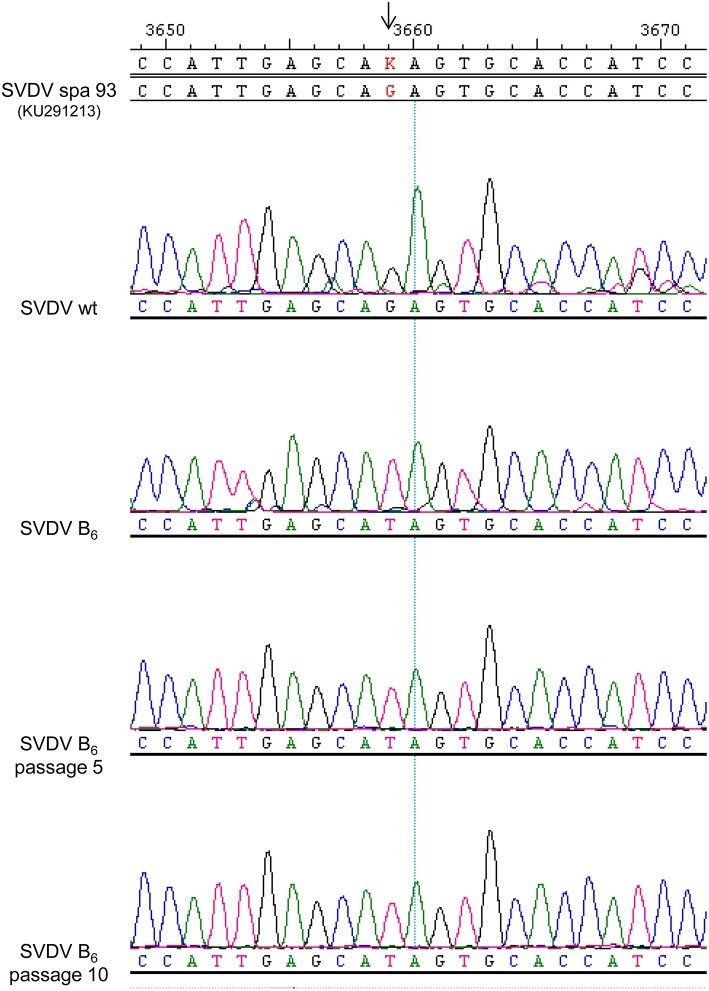
**Genetic stability of the nucleotide replacement G3659T**. SVDV B_6_ was passaged ten times in absence of BFA. The viral RNA of the passages 5 and 10 was extracted and the nucleotide sequence was determined. Representative chromatograms of the region encoding 2C protein are shown of SVDV wt, SVDV B_6_, SVDV B_6_ passage 5 and SVDV B_6_ passage 10.

The comparison of 86 complete 2C protein sequences of different enterovirus retrieved from a BLAST analysis (Mount, 2007) showed that residue Q65 is invariant in all the viruses compared (Table 3). To test the stability of the nucleotide substitution G3659T responsible for replacement Q65H, the SVDV B_6_ population was serially passaged ten times in the absence of BFA. The substitution G3659T was conserved in the average population after these passages, confirming its genetic stability (Figure 5).

**Table 3:** **Representative multiple sequence alignment of 2C proteins from different enterovirus and the SVDV strain used in this work (SVDV SPA/1/′93)**.

**Virus**	**Amino acid sequence of 2C protein**	**GenBank accesion**
SVDV spa93	NSGWLKKFTEMTNACKGMEWIAIKIQKFIEWLKVKILPEVKEKHEFLNRLKQLPLLESQIATIEQSAPSQGDQEQLFSNVQYFAHYCRKYAPLYAAEAKR	KU291213
SVDV Itl	NSGWLKKFTEMTNACKGMEWIAIKIQKFIEWLKVKILPEVKEKHEFLNRLKQLPLLESQIATIEQSAPSQGDQEQLFSNVQYFAHYCRKYAPLYAAEAKR	EU151461.1
CVB5	NNGWLKKFTEMTNACKGMEWIAVKIQKFIEWLKVRILPEVKEKHEFLNRLKQLPLLESQIATIEQSAPSQGDQEQLFSNVQYFAHYCRKYAPLYAAEAKR	CCW33411.1
CVB4	NNSWLKKFTEMTNACKGMEWIAVKIQKFIEWLKVRILPEVKEKHEFLNRLKQLPLLESQIATIEQSAPSQSDQEQLFSNVQYFAHYCRKYAPLYAAEAKR	CCW33464.1
CVB3	NNGWLKKFTEMTNACKGMEWIAIKIQKFIEWIKVKILPEVKEKHEFLNRLKQLPLLESQIATIEQSAPSQSDQEQLFSNVQYFAHYCRKYAPLYATEAKR	CCW33459.1
CVB2	SNGWLKKFTEMTNACKGMEWIAVKIQKFIEWLKVKILPEVKEKHEFLNRLKQLPLLESQIATIEQSAPSQSDQEQLFSNVQYFAHYCRKYAPLYATEAKR	CCW33458.1
CVA9	NNGWLKKFTEMTNACKGMEWIAIKIQKFIEWLKVKILPEVREKHEFLNRLKQLPLLESQIATIEQSAPSQSDQEQLFSNVQYFAHYCRKYAPLYAAEAKR	CCW33456.1
EVB83	NNNWLKKFTEMTNACKGMEWIAVKIQKFIEWLKVKILPEVKEKHEFLNRLKQLPLLESQIATIEQSAPSQSDQEQLFTNVQYFAHYCRKYAPLYAAEAKR	CCW33489.1
EV13	NNGWLKKFTEMTNACKGMEWIAIKIQKFIEWLKVKVLPEVKEKHEFLNRLKQLPLLESQIATIEQSAPSQSDQEQLFSNVQYFAHYCRKYAPLYATEAKR	AAX63470.2
ECHO30	NNGWLKKFTEMTNACKGMEWIAIKIQKFIEWLKVKILPEVREKHEFLNRLKQLPLLESQIATIEQSAPSQSDQEQLFSNVQYFAHYCRKYAPLYAAEAKR	AAS83471.3
ECHO21	NNGWLKKFTEMTNACKGMEWIAIKIQKFIEWLKVKILPEVREKHEFLNRLKQLPLLESQIATIEQSAPSQSDQEQLFSNVQYFAHYCRKYAPLYAAEAKR	CCW33484.1
ECHO25	NNGWLKKFTEMTNACKGMEWIAIKIQKFIEWLKVKIMPEVKEKHEFLNRLKQLPLLESQVATIEQSAPSQSDQEQLFSNVQYFAHYCRKYAPLYAAEAKR	CCW33486.1
ECHO6	NNGWLKKFTEMTNACKGMEWIAIKIQKFIEWLKVKILPEVREKHEFLNRLKQLPLLESQIATIEQSAPSQSDQEQLFSNVQYFAHYCRKYAPLYAAEAKR	CBL42967.1
ECHO33	NNGWLKKFTEMTNACKGMEWIAIKIQKFIEWLKVKILPEVKEKHEFLNRLKQLPLLESQITTIEQSAPSQSDQEQLFSNVQYFAHYCRKYAPLYAAEAKR	CCW33488.1
ECHO2	NSGWLKKFTEMTNACKGMEWIAIKIQKFIEWLKVKILPEVKEKHEFLNRLKQLPLLESQIATIEQSAPSQSDQEQLFSNVQYFAHYCRKYAPLYAAEAKR	CCW33466.1
ECHO14	NSGWLKKFTEMTNACKGMEWIAIKIQKFIEWLKVKILPEVKEKHEFLNRLKQLPLLESQIATIEQSAPSQSDQEQLFSNVQYFAHYCRKYAPLYAAEAKR	CCW33478.1
ECHO18	NSGWLKKFTEMTNACKGMEWIAIKIQKFIEWLKIKILPEVKEKHEFLNRLKQLPLLESQIATIEQSAPSQSDQEQLFSNVQYFAHYCRKYAPLYAAEAKR	CCW33479.1
ECHO3	NSGWLKKFTEMTNACKGMEWIAIKIQKFIEWLKIKILPEVKEKHEFLNRLKQLPLLESQIATIEQSAPSQSDQEQLFSNVQYFAHYCRKYAPLYAAEAKR	CCW33467.1
PV	GDSWLKKFTEACNAAKGLEWVSNKISKFIDWLKEKIIPQARDKLEFVTKLRQLEMLENQISTIHQSCPSQEHQEILFNNVRWLSIQSKRFAPLYAVEAKR	ACX85868.1
	^* * * * * * *^ ^* *^ ^* *^ ^* *^ ^**^ ^***^ ^* * *^ ^** *^ ^*^ ^**^ ^* **^ ^**^ ^** **^ ^* *^ ^* *^ ^* *^ ^***^ ^* *^ ^*****^ ^* * * *^	

Residues 1–100 are shown in the figure. The residues found replaced in SVDV br1-br9 BFA resistant viruses are highlighted: R49 in red and Q65 in green. Residues invariant in the alignment are indicated by ^*^.

### BFA-resistant SVDV br1 induces golgi disassembly

**Figure 6:**
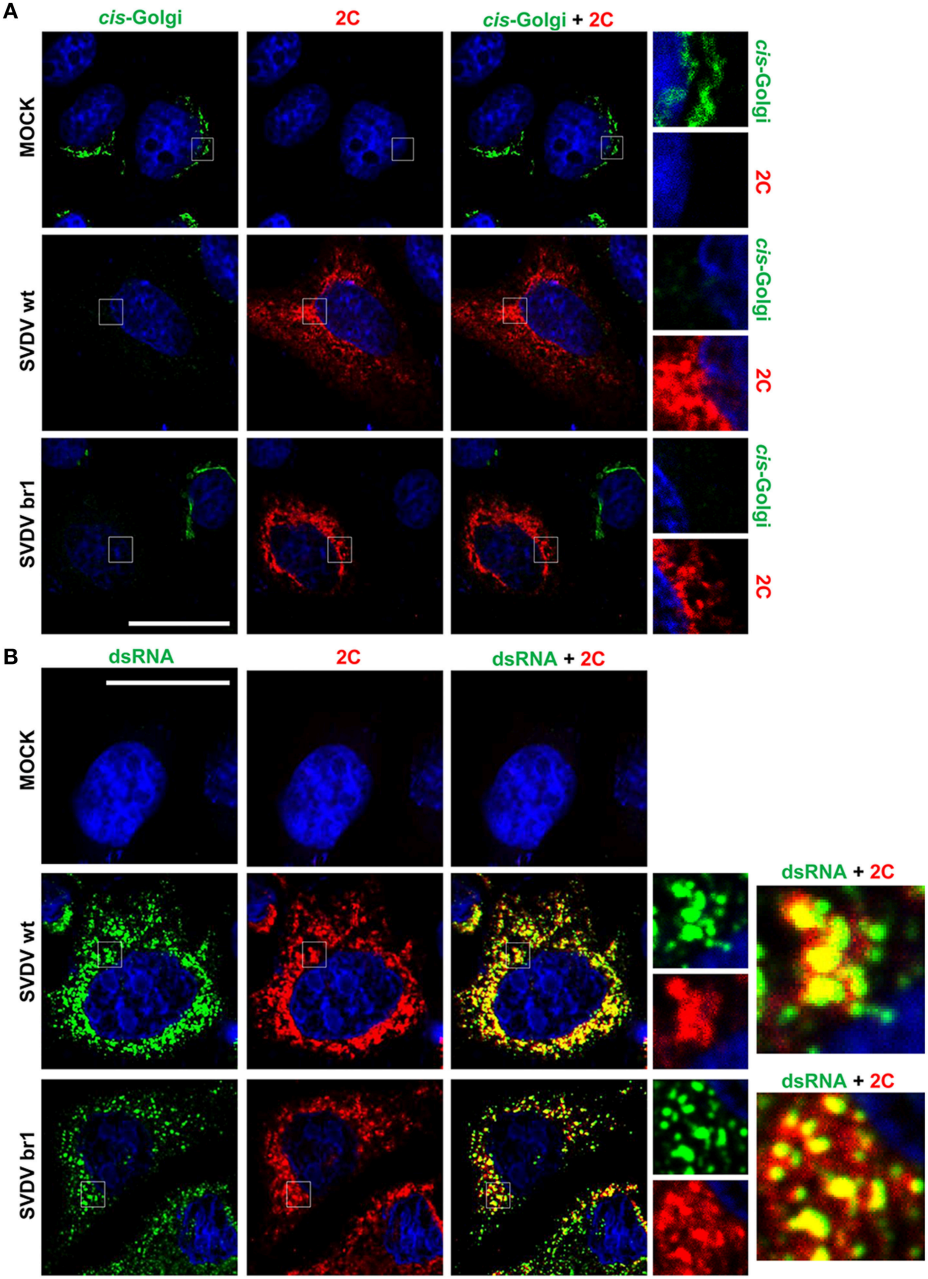
**Effect of the infection on Golgi complex and cellular localization of viral protein 2C. (A)** IBRS-2 cells infected with SVDV wt or br1 (moi of 0.5 pfu/cell) and processed for double immunofluorescence, at 8 h pi, using MAb to GM130 (*cis*-Golgi) or a rabbit polyclonal serum to 2C protein. **(B)** IBRS-cells infected and processed for immunofluorescence as in **(A)** using MAb to dsRNA or a rabbit polyclonal serum to 2C protein. The right panels represent the detail inside the box. Nuclei were stained using Topro-3. Bar: 20 μm.

SVDV infection of IBRS-2 cells disassembles ER and Golgi complex. In addition to this, the viral protein 2C is mainly located perinuclearly whit a punctuated pattern labeling the viral replication complexes (Martín-Acebes et al., 2008). In order to study whether the membrane rearrangements produced by BFA-resistant SVDV were different from those of SVDV wt, IBRS-2 cells were infected with SVDV wt or br1 and processed for immunofluorescence analysis (Figure 6). SVDV br1 was selected for these experiments because this mutant only differs from the wt on the amino acid replacement 2C Q65H (Table 2). SVDV br1 infection disassembled the *cis*-Golgi as occurred with the wt infection (Figure 6A). Moreover, the perinuclear localization of 2C and its distribution pattern was indistinguishable from that of wt SVDV (Figure 6). In addition, 2C colocalized with the dsRNA fluorescence in both wt and br1 SVDV-infected cells (Figure 6B). These results indicate that replication of the BFA-resistant SVDV induces Golgi disassembly and does not display remarkable differences with the wt virus in the cellular membranes rearrangements produced and the viral replication complex localization.

### The amino acid substitution Q65H in the SVDV 2C protein also confers resistance to GCA

**Figure 7:**
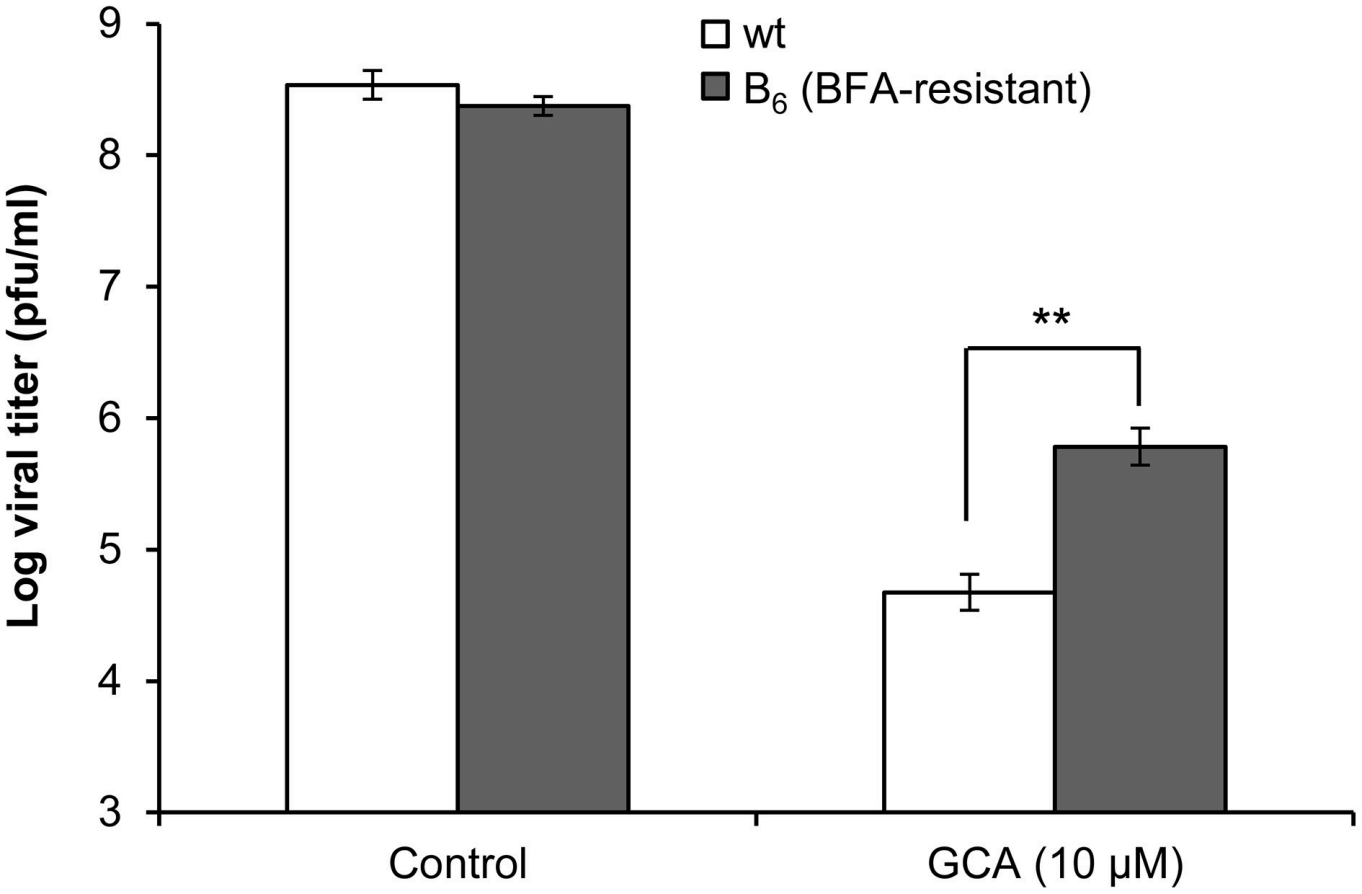
**The amino acid substitution Q65H in 2C confers resistance to GCA**. IBRS-2 cells treated with 10 μM GCA for 30 min were infected with SVDV wt or SVDV B_6_ (moi of 0.5 pfu/cell) in presence of the drug. At 8 h pi the viral titer was determined by plaque assay. Error bars represent SD. Data were analyzed using ANOVA. Two asterisks (^**^) denote statistically significant differences with *P* < 0.005.

The production of SVDV was inhibited by BFA as well as by the Golgi disrupting agent GCA (Figure 1C). To test whether the SVDV B_6_ population was also resistant to GCA, IBRS-2 cells were infected with SVDV wt or B_6_ in presence of 10 μM GCA and the viral titer was determined 8 h pi (Figure 7). These experiments showed an increase in the resistance to GCA of SVDV as its multiplication was inhibited about 1 order of magnitude less in the presence of GCA than that of SVDV wt. These results indicate that replacement 2C Q65H is responsible for the resistance to agents that inhibit GBF1.

## Discussion

The study of the molecular factors usurped by viruses in their cellular hosts provides valuable information to understand virus-host interactions. Picornaviruses reorganize the cellular membranes of the infected cells to generate specialized organelles for viral RNA replication, but how this process occurs is not yet fully understood. SVDV infection disassembles the ER and the Golgi complex and the viral replication takes place at the surface of cytoplasmic vesicle-like structures likely originated from these organelles (Martín-Acebes et al., 2008). However, the differences in the morphology of cytoplasmic rearrangements induced in infected cells and in the sensitivity to Golgi-disrupting agents, like BFA or GCA, suggest the existence of diverse cellular requirements among picornaviruses to form the replication complex (Moffat et al., 2005; Wessels et al., 2006a; Martín-Acebes et al., 2008; van der Linden et al., 2010). In fact, there is evidence of important differences on host factors involved in the replication of the picornaviruses, such as COP-I (Gazina et al., 2002), Arf1 (Midgley et al., 2013; Wang et al., 2014b), ACBD3 (acyl-CoA binding domain containing 3) (Greninger et al., 2012) or the recruitment of phosphatidylinositol 4-kinase class III β (PI4KIIIβ) (Sasaki et al., 2012; Dorobantu et al., 2014, 2015).

In the present study, we have documented the sensitivity of SVDV infection to BFA and GCA, two different drugs that target the secretory pathway. BFA inhibits GBF1, BIG1 and BIG2; three members of a family of GEFs that activate several small Arf GTPases (Belov and Sztul, 2014), while GCA is a specific inhibitor of GBF1 (Sáenz et al., 2009; Belov and Sztul, 2014). The effect of BFA and GCA on SVDV production was similar, which could indicate that, as proposed for PV and CVB3, the sensitivity of SVDV infection to BFA inhibition is dependent on GBF1, but not on BIG1/2 (Belov et al., 2008; Lanke et al., 2009; van der Linden et al., 2010). Furthermore, the BFA-resistant SVDVs here described also displayed increased resistance to GCA, supporting that the molecular basis of BFA resistance is related to GBF1, which is specifically inhibited by GCA. Overall, our results suggest that the inhibition on SVDV replication by BFA and GCA relies on its effect on GBF1. In fact, GBF1 variants unable to activate Arf1 can partially rescue enterovirus replication from BFA inhibition, which is also consistent with the view that GBF1 actually plays a role in enterovirus replication regardless its ability to activate Arf1 (Belov et al., 2010). On the other hand, while PV mutants resistant to BFA can be selected in Hela cells, the isolation process was not possible in Vero cells as a consequence of the lower availability of GBF1 for the formation of the replication complex in this cell line (Viktorova et al., 2015). Moreover, studies performed with Aichi virus, another picornavirus not sensitive to BFA, showed that the replication of this virus is strongly inhibited by a siRNA-mediated knockdown of GBF1 (Greninger et al., 2012), further supporting a role of GBF1 on picornavirus replication independently of its ability to activate Arf1. The results presented here are similar to those reported for other enterovirus such as PV and CVB3 (Lanke et al., 2009; Hsu et al., 2010; Viktorova et al., 2015), which suggest that GBF1 could be also localized in the viral replication factories of SVDV. Nevertheless, there is no direct evidence that GBF1 forms part of the SVDV replication complex and further work is required to ascertain this point.

Our analysis revealed that the molecular basis for the BFA-resistant SVDV here described was related to a single nucleotide substitution responsible for amino acid replacement Q65H in non-structural protein 2C. Picornavirus 2C is a conserved protein (329 amino acids in length for SVDV) with a hydrophobic N-terminal domain that mediates its binding to membranes (Echeverri and Dasgupta, 1995; Teterina et al., 1997a; Echeverri et al., 1998). In the case of SVDV, 2C protein colocalizes with the viral replication sites (Martín-Acebes et al., 2008), in a parallel manner to that described for other enteroviruses (Springer et al., 2013), and it is involved in PV membrane rearrangements (Teterina et al., 1997b). Moreover, 2C plays a role in PV RNA synthesis (Barton and Flanegan, 1997; Smithee et al., 2015) and virion packaging (Li and Baltimore, 1990; Liu et al., 2010; Wang et al., 2014a). Substitution Q65H does not provoke a major impact on the cellular distribution of 2C protein, since both wt and BFA-resistant SVDV exhibited a similar fluorescent pattern which was associated to viral replication complexes labeled with dsRNA. Studies performed with PV 2C protein have revealed that it contains a N-terminal domain amphipathic helix, two predicted RNA-binding regions, three motifs involved in NTP binding, and a cysteine-rich putative zinc finger motif (Springer et al., 2013). Although the 3D structure of 2C protein has not been solved for any picornavirus, the secondary structure prediction of SVDV 2C protein using the Phyre engine (Bennett-Lovsey et al., 2008) located the residue Q65 immediately after an alpha helix motif, relatively close to the membrane binding domain (residues 6–54 in the PV 2C protein) (Echeverri and Dasgupta, 1995; Teterina et al., 1997a; Echeverri et al., 1998). As commented above, other groups have previously described PV mutants resistant to BFA carrying point mutations in 2C and 3A proteins (Crotty et al., 2004; Viktorova et al., 2015). In these cases, the resistance to BFA was mainly associated with amino acid replacements in the 3A, whereas the substitution in the 2C seemed to have an auxiliary role. Indeed, PV 2C and 3A exhibit functional interactions during viral replication, a direct binding between these proteins has been reported (Teterina et al., 2006), and mutations in one protein can be compensated by changes in the other (Teterina et al., 2006). To our knowledge, there is no evidence of a direct interaction between enterovirus 2C protein and GBF1, although it has been demonstrated that the enteroviral protein 3A interacts directly with GBF1 (Wessels et al., 2006a,b, 2007). Having in mind all these considerations, the molecular basis of the increase in resistance to BFA observed for our SVDV mutants could rely on an alteration on their ability to interact with GBF1, either directly or through its interaction with 3A protein.

In general, antiviral agents can interfere with viral components in processes that often lead to development of drug resistance in virus populations evolving under selective pressures (Domingo and Gomez, 2007; Colman, 2009). One of the approaches to evade this limitation is the identification of antivirals targeted against cellular functions required for the virus to complete its viral cycle (Khattab, 2009; Ludwig, 2009; Pereira and Jacobson, 2009). Despite BFA targeting a cellular protein, our results show that the inhibition of SVDV replication exerted by BFA can be bypassed by the selection of drug-resistance virus mutants. This is in agreement with results obtained by other authors using PV (Crotty et al., 2004; Viktorova et al., 2015), although in our results with SVDV, drug resistance is mainly associated with changes in 2C protein. These results also unveil a functional connection between GBF1 protein and 2C protein in SVDV replication, thus providing further evidence of the involvement of GBF1 in the replication of this pathogen.

## Author contributions

Conceived and designed the experiments: AV-C, MAM-A, FS. Performed the experiments: AV-C, FC, MG-M, MAM-A. Analyzed the data: AV-C, MAM-A, FS, J-CS. Contributed to the writing if the manuscript: AV-C, MAM-A, FS, J-CS.

## Funding

This work was supported by grants BIO2011-24351 and AGL2014-52395-C2-1-R (FS) from MINECO, AGL2014-56518-JIN (MAM-A) from MINECO, and by RTA2013-C0013-E from INIA and partially financed by the European Regional Development's funds (FEDER) and S2013/ABI-2906-PLATESA from CAM and partially financed by FEDER (FS and J-CS). Work at Centro de Biología Molecular “Severo Ochoa” was also supported by Fundación Ramón Areces. AV-C is a recipient of a “Contrato de formación postdoctoral” from MINECO. The funders had no role in study design, data collection and interpretation, or the decision to submit the work for publication.

### Conflict of interest statement

The authors declare that the research was conducted in the absence of any commercial or financial relationships that could be construed as a potential conflict of interest.
